# Comparative Anatomy of the Hyoid Apparatus in Various Dog Breeds: Insights From Clinical Imaging

**DOI:** 10.1002/vms3.70932

**Published:** 2026-04-10

**Authors:** Yusuf Altundağ, Ermiş Ozkan, Funda Yiğit, Kozet Avanus, Nedzad Hadziomerovic, Tomasz Szara, Constantin Spataru

**Affiliations:** ^1^ Department of Surgery, Faculty of Veterinary Medicine Namik Kemal University Tekirdag Türkiye; ^2^ Department of Anatomy, Faculty of Veterinary Medicine Istanbul University‐Cerrahpasa Istanbul Türkiye; ^3^ Department of Histology and Embryology, Faculty of Veterinary Medicine Istanbul University‐Cerrahpaşa Istanbul Türkiye; ^4^ Department of Animal Breeding and Genetics, Faculty of Veterinary Medicine Istanbul University‐Cerrahpaşa Istanbul Türkiye; ^5^ Department of Basic Sciences of Veterinary Medicine, Veterinary Faculty University of Sarajevo Sarajevo Bosnia and Herzegovina; ^6^ Department of Morphological Sciences, Institute of Veterinary Medicine Warsaw University of Life Sciences Warsaw Poland; ^7^ Department of Preclinics, Faculty of Veterinary Medicine Iasi University of Life Sciences Iasi Romania

**Keywords:** anatomy, carnivore, geometric morphometrics, stylohyoid, thyrohyoid, veterinary surgery

## Abstract

**Background:**

Studying the hyoid bone in dogs is of significant importance in veterinary surgery and anatomy, as it aids in understanding how variations in this structure may affect tongue mobility, swallowing and vocalisation across breeds.

**Objectives:**

This study aims to provide preliminary insights into the relationship between structural differences in the hyoid bone and breed‐specific functional adaptations in tongue shape and movement by analysing shape variations within the hyoid apparatus.

**Methods:**

Hyoid bones from computed tomography images of 26 dogs were modelled, and principal component analysis (PCA) was conducted to examine shape variation in the hyoid bones. Additionally, the influences of age and weight on hyoid bone shape were assessed.

**Results:**

PCA showed that PC1 (42.4%) reflected a relatively conservative pattern related to hyoid and skull morphology, whereas PC2 and PC3 indicated greater individual variation. Brachycephalic breeds exhibited a more dorsoventrally positioned and compact hyoid structure, while mesocephalic breeds showed a more aligned and elongated configuration of the stylohyoid and thyrohyoid bones. No significant correlations were found between hyoid shape and age, weight or Procrustes distance, suggesting a stronger influence of genetic factors.

**Conclusions:**

Understanding the morphological variation of the hyoid bone in dogs contributes to veterinary anatomy and has practical applications in veterinary medicine, particularly in surgical and rehabilitation practices. Given the hyoid apparatus's critical role in swallowing and vocalisation, insights from this study may enhance clinical approaches to treating conditions linked to hyoid bone morphology.

## Graphical abstract

This study identifies breed‐related shape differences in the canine hyoid apparatus using geometric morphometrics. The results suggest that skull type influences hyoid morphology, with potential functional implications for swallowing, vocalisation and tongue movement.

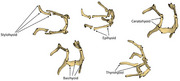

## 1 Introduction

The hyoid apparatus in dogs is a complex structure consisting of several small bones located in the throat, near the base of the tongue (Evans and De Lahunta 2012). This system is essential for supporting and moving the tongue and is also involved in the function of the laryngeal muscles (Takada et al. 2009). In dogs, as in other placental mammals, the hyoid apparatus includes a central bar called the basihyoid or body, positioned transversely at the front of the larynx. Two sets of projections, or cornua, are extended from the basihyoid: the lesser cornua and the greater cornua. The lesser cornua form the longer front pair, while the greater cornua are the shorter rear pair. The naming discrepancy reflects the human structure, where the greater cornua are larger, and the lesser cornua exist only as the ceratohyoid (Kealy et al. 2010). Each lesser cornu comprises four small bones—the ceratohyoid, epihyoid, stylohyoid and tympanohyoid—which curve forward and upward to connect with the skull (Weissengruber et al. 2002). The tympanohyoid attaches to the temporal bone through a ligamentous connection. On each side, the greater cornu is formed by the thyrohyoid, which joins with the thyroid cartilage of the larynx. A thorough understanding of the typical structure and function of the canine hyoid apparatus is important for clinicians and researchers working on various tongue‐related disorders and syndromes.

Three‐dimensional (3D) modelling and shape analysis have become essential in anatomical and biological studies, allowing researchers to investigate structural diversity and the functional significance of anatomical features across species and within populations (Gündemir et al. 2023a; Toryan et al. 2024; Bakici et al. 2021; Duro et al. 2021; Klingenberg 2016). This method goes beyond traditional size‐based measurements, enabling a more detailed exploration of morphological variation by capturing subtle differences in form (Kocak et al. 2024; Manuta et al. 2024; Özkan et al. 2024). In particular, geometric morphometrics—a specialised technique in shape analysis—uses defined anatomical landmarks to record precise shape information, facilitating statistical comparisons and visual representation of shape differences (Bookstein 2023; Gündemir et al. 2024; Korkmazcan et al. 2025). This approach has broad applications in veterinary anatomy, where it is used to study species‐specific adaptations, breed‐related morphology and the implications of these variations on animal health and function (Jashari et al. 2022; Gündemir et al. 2023b; Szara et al. 2023). By applying geometric morphometrics, researchers in veterinary anatomy can examine how shape variations relate to the mechanical demands and environmental influences that shape each species or breed (Güzel et al. 2024; Manuta et al. 2023; Hadžiomerović et al. 2023).

Studying the hyoid bone in dogs holds significant value for veterinary surgery and anatomy, as it helps to understand how changes in this structure may impact tongue mobility, swallowing and vocalisation across breeds. The hyoid apparatus, located at the base of the tongue, is critical in supporting and controlling tongue movement, influencing feeding, respiratory function and communication behaviours in dogs. This study aims to gain preliminary insights into how structural differences in the hyoid bone relate to breed‐specific functional adaptations in tongue shape and movement by analysing shape variations in the hyoid apparatus. Additionally, weight and age will be considered to assess their potential effects on hyoid morphology and examine whether these parameters impact the hyoid bone. Such findings may provide valuable information for veterinary clinicians and anatomists, illuminating breed‐specific anatomical features and informing medical, surgical and rehabilitative practices in canine care.

## 2 Material and Methods

### 2.1 Ethical Statement

This study was approved by the Istanbul University‐Cerrahpaşa, Animal Experiments Local Ethics Committee.

### 2.2 Animals and Modelling

In this investigation, hyoid bones from 26 dogs were analysed. The age range, weight, sex and breed of the dogs involved in the study are recorded (Table 1). The images of the dogs were sourced from the Istanbul University‐Cerrahpaşa, Faculty of Veterinary Medicine, Research and Practice Animal Hospital. All dogs included in the study were free of orthopaedic or pathological disorders affecting the skull, including the mandible. The cephalic type of each dog was determined by calculating the cephalic index, which was measured as the ratio of skull width to skull length following standard morphometric methodology. Computed tomography (CT) scan was performed using a 4‐slice multidetector CT scanner (Siemens Somatom Scope, vc30b). The scanning parameters were set to a slice thickness of 0.6 mm, with 110 kV and 28 mA, completing the scan in 14 s. The resulting images were stored in DICOM format, and 3D modelling operations were conducted using Slicer software (version 5.2.2; Bakici et al. 2019a; Rolfe et al. 2021). Prior to 3D reconstruction, multiplanar reconstruction (MPR) was applied to the CT images to ensure accurate orientation and identification of the hyoid elements. During the modelling process, soft tissues and the skull and mandible were removed from the images. The processed images were then saved in ‘ply’ format.

**TABLE 1:** Samples.

No.	Breed	Sex	Age (year)	Weight (kg)
1	Bouvier De Flandre	Male	14	34
2	Chihuahua	Male	9	3
3	English Setter	Female	2	15
4	French Bulldog	Male	5	16
5	French Bulldog	Female	7	14
6	German Shepherd	Male	5	30
7	German Shepherd	Male	7	28
8	German Shepherd	Male	9	34
9	Golden Retriever	Male	7	40
10	Golden Retriever	Male	8	33
11	Golden Retriever	Female	10	32
12	Golden Retriever	Male	12	33
13	Golden Retriever	Female	14	22
14	Jack Russel	Male	6	8
15	Kangal Shepherd	Female	2	25
16	Mix	Male	6	30
17	Mix	Female	9	22
18	Mix	Male	10	40
19	Mix	Male	13	35
20	Pincher	Female	5	4
21	Pug	Male	4	12
22	Rottweiler	Female	5	25
23	Terrier	Male	10	11
24	Terrier	Female	12	13
25	Yorkshire Terrier	Male	9	3
26	Yorkshire Terrier	Male	11	3

All CT images were initially reviewed by veterinary diagnostic imaging specialists to confirm the absence of any pathological conditions affecting the skull, including the hyoid region. Following this, the 3D reconstruction and landmarking processes were conducted by the same experienced researcher to ensure standardisation and consistency across all samples.

### 2.3 Landmarks

**FIGURE 1:**
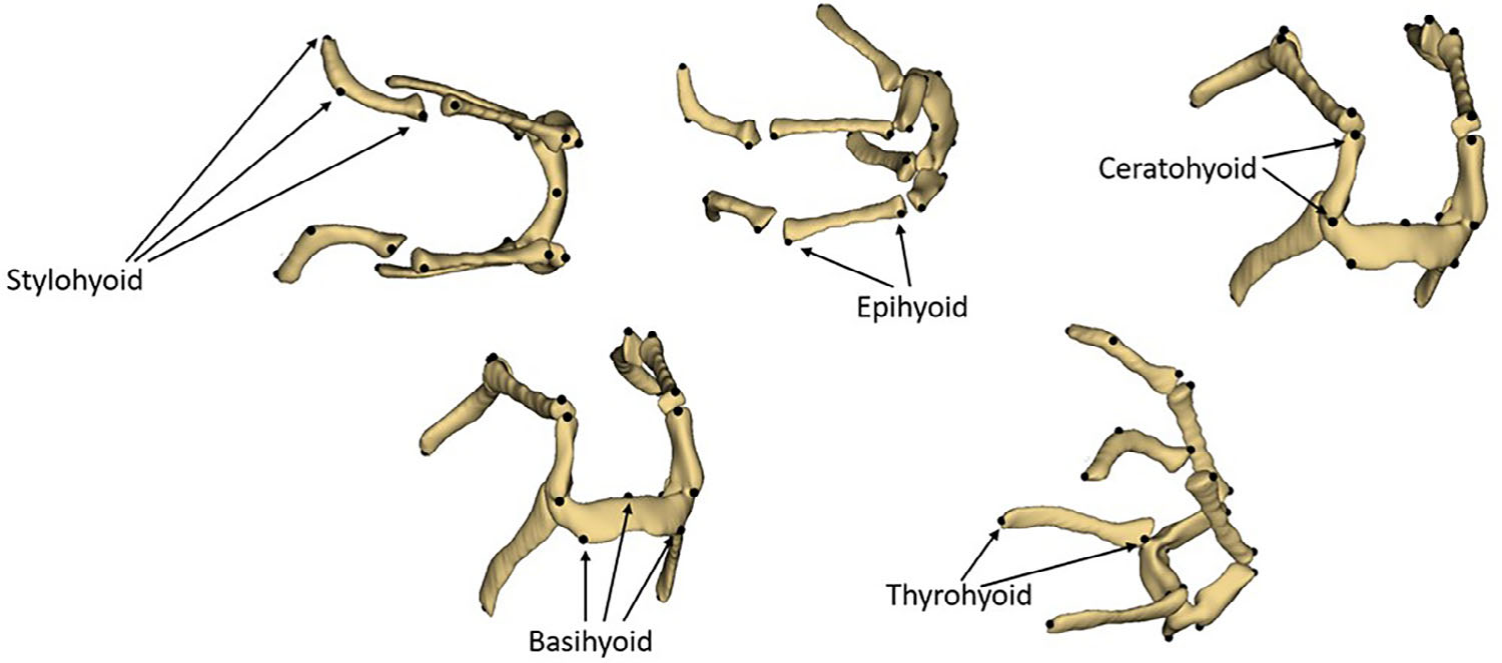
Landmarks: three landmarks for stylohyoid, two landmarks for epihyoid, two landmarks for ceratohyoid, three landmarks for basihyoid and two landmarks for thyrohyoid.

A total of 21 landmarks were selected, and the Slicer software (version 5.2.2) was used for the landmarking process. Initially, a draft set of landmarks was created for the hyoid bones, with all 21 landmarks successfully applied to each sample and no missing data in the analysis. Three landmarks were designated for the stylohyoid, two for the epihyoid, two for the ceratohyoid, three for the basihyoid and two for the thyrohyoid. The tympanohyoid was excluded from the analyses as it was not fully captured in all images (Figure 1).

The selection of landmarks was based on clear anatomical reference points that are consistently identifiable across all specimens and that represent key structural components of the hyoid apparatus. Landmarks were chosen to reflect functionally and morphologically significant regions, such as articulation points, bone junctions and tips of individual elements (stylohyoid, epihyoid, ceratohyoid, basihyoid and thyrohyoid). These points were selected to capture shape variation effectively while ensuring reproducibility and minimising observer error.

### 2.4 Geometric Morphometrics and Statistical Analysis

Centroid size data were collected for each group of dog hyoid bones, yielding the total size (centroid size) for each bone set. Principal component analysis (PCA) was then employed to explore shape differences in the hyoid bones (Boz et al. 2023). PCA identified patterns within complex datasets by transforming correlated variables (landmark coordinates) into orthogonal components called principal components (PCs). PC1, PC2 and PC3 were identified as the components explaining the most variation in hyoid bone shape and were considered sufficient to reveal significant shape variations. Visual representations, such as graphs, were used to illustrate the distribution of individuals across PC1, PC2 and PC3.

To evaluate the effect of size and other biological variables on shape, multivariate regression analysis was performed using PAST (V 4.14) software with PC1 as the dependent variable. Independent variables included centroid size, body weight and age. Significance was determined at a threshold of *p* < 0.05.

## 3 Results

PCA conducted on the hyoid bone data resulted in the identification of a total of 10 PCs. However, only the first three PCs were deemed significant for evaluation, as they collectively accounted for over 10% of the total variation within the dataset. Specifically, PC1 explained 42.4% of the variation, indicating that this component captures the most substantial differences in hyoid bone morphology among the studied dog breeds. PC2 and PC3 accounted for 17.8% and 13.3% of the variation, further elucidating additional shape characteristics.

**FIGURE 2:**
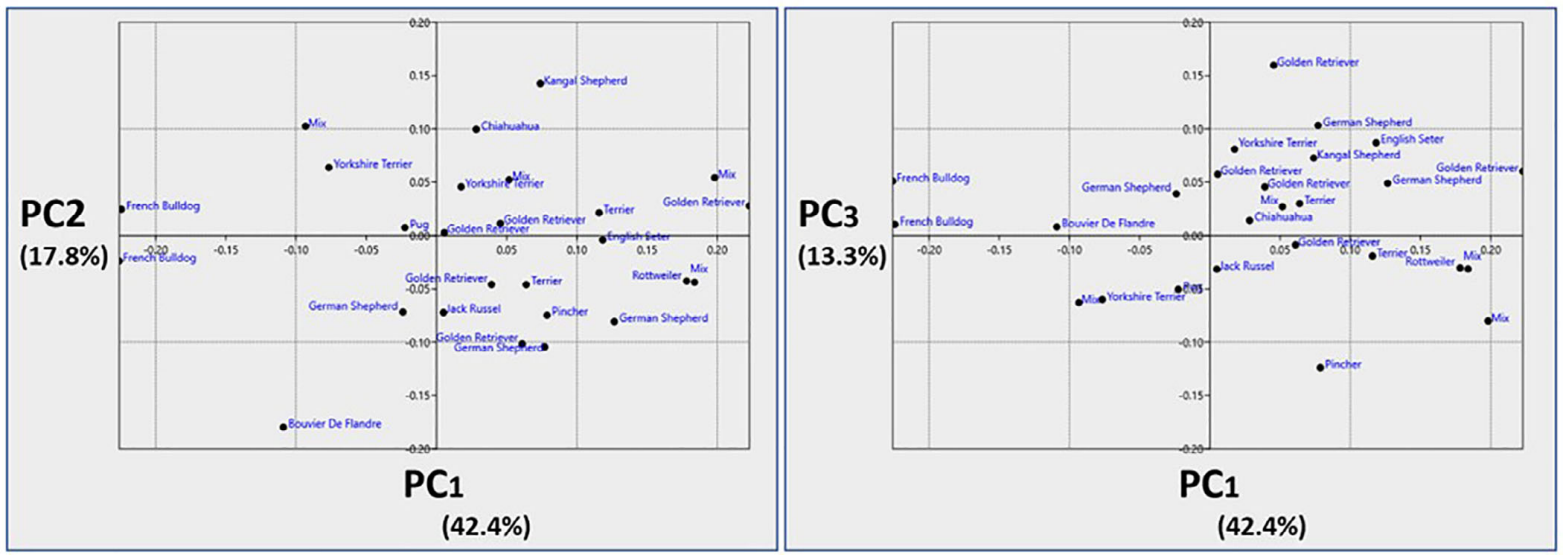
Principal component analysis scatter plot of dog breeds (PC1, PC2 and PC3).

The distribution of individuals across these PCs is visually represented in Figure 2. PC1 explained 42.4% of the total shape variation and primarily reflected changes in the position and compactness of the hyoid apparatus. Negative PC1 values were associated with a shorter and more dorsoventrally compact hyoid structure, where the basihyoid was positioned closer to the skull base. This morphology was characteristic of brachycephalic breeds such as French Bulldogs and Pugs. In contrast, positive PC1 values indicated a more elongated and anteriorly extended hyoid apparatus, as observed in breeds like Golden Retrievers and German Shepherds.

Interestingly, all Golden Retrievers were consistently associated with positive PC1 values, which could imply a unique hyoid morphology linked to their breed characteristics.

Additionally, breeds such as Terriers and German Shepherds displayed a broader variation along PC1.

PC2 accounted for 17.8% of the variation and reflected the degree of separation between the stylohyoid and thyrohyoid bones. Negative PC2 values showed a closer alignment and compact arrangement between these bones, while positive PC2 values reflected a more separated configuration.

PC3 explained 13.3% of the variation and mainly captured changes in the shape and size of the ceratohyoid bone. Positive PC3 values were characterised by an enlarged and more prominent ceratohyoid, indicating an anterior shape shift in the hyoid apparatus.

While the distribution along PC1 appeared to show a more conservative pattern related to the morphology of the hyoid bone and skull structures, the distributions for PC2 and PC3 revealed more significant individual variation. No distinct morphological patterns representing specific traits were observed among the different breeds in this study.

**FIGURE 3:**
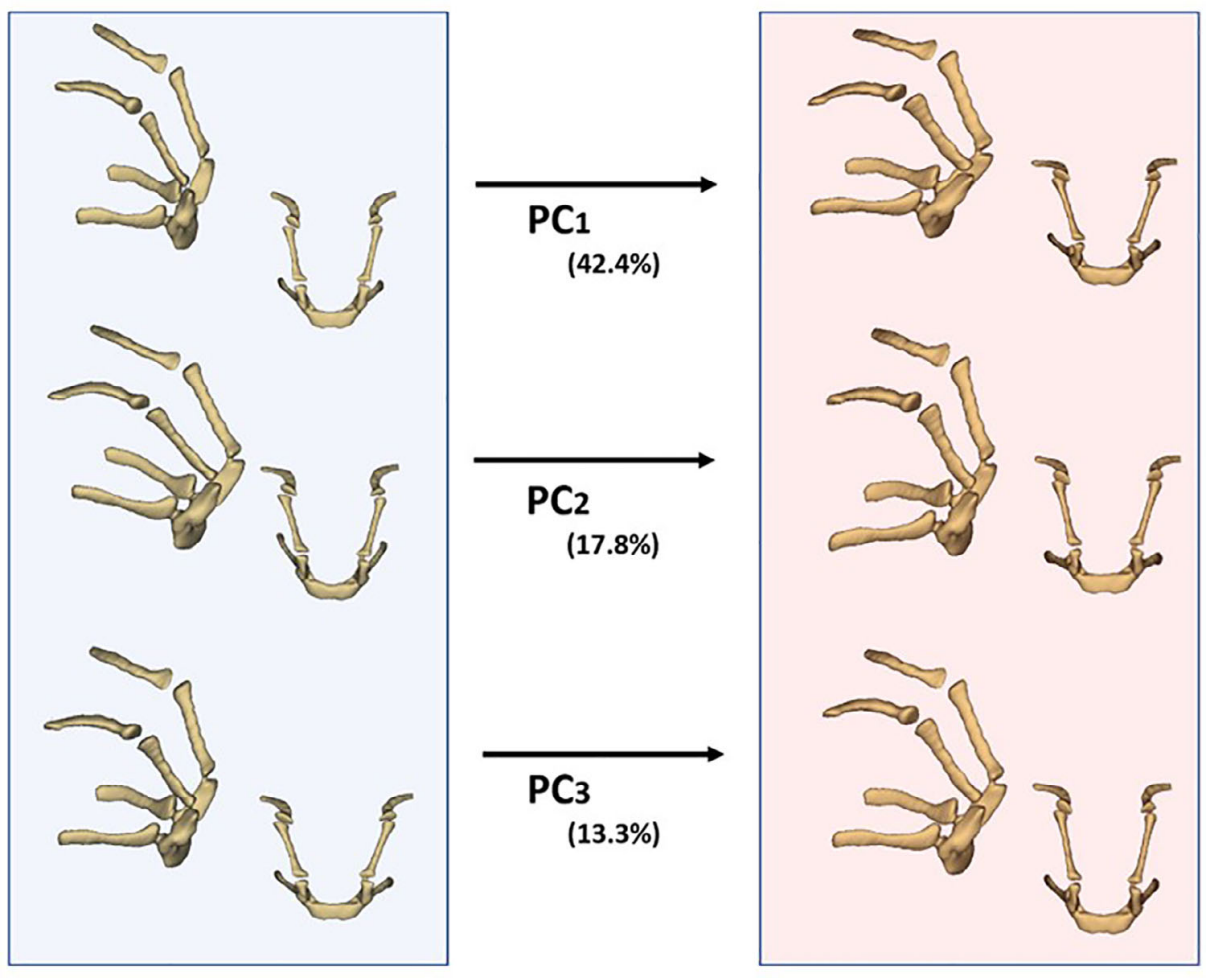
Comparison of hyoid bone shape variations between negative (blue) and positive (red) values of PC1, PC2 and PC3 from lateral and dorsal views.

The shape changes represented by PC1 indicate a significant variation in the morphology of the hyoid bones, transitioning from a narrower configuration to a more elongated structure (Figure 3). Specifically, negative PC1 values correspond to a more pronounced hyoid apparatus, where the basihyoid is positioned closer to the base of the skull.

In contrast, positive PC1 values indicate a more anteriorly positioned basihyoid. In positive PC1 values, the stylohyoid and thyrohyoid also showed a more lateral positioning.

Moving on to PC2, the negative values revealed that the stylohyoid and thyrohyoid bones are closely aligned in structure, forming a more compact arrangement. Conversely, positive values of PC2 indicated that the stylohyoid and thyrohyoid are positioned farther apart. Finally, PC3 highlighted significant anterior shape variations, particularly in the ceratohyoid. In positive values of PC3, the ceratohyoid exhibited a larger structure, making it the most prominent bone among the hyoid elements.

PC1 was selected for analysis to assess the allometric effects on the hyoid bones, as it accounted for the most significant variation observed in the dataset. The remaining PCs, PC2 and PC3 explained comparatively less variation and exhibited a more individualised distribution of shape characteristics among the dog breeds.

**FIGURE 4:**
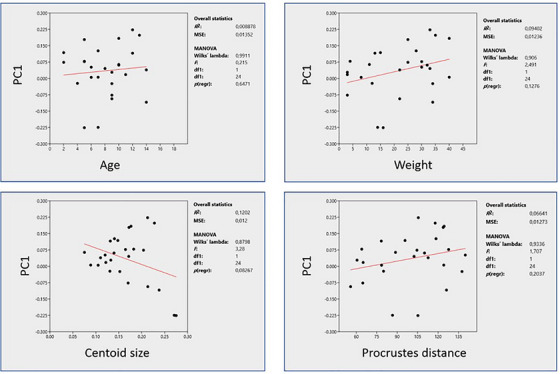
Allometry for hyoid bones.

The analysis demonstrated that centroid size emerged as the most significant measurement influencing shape variation along PC1, as depicted in Figure 4. A negative correlation was observed between centroid size and PC1, although this correlation was statistically insignificant.

Additionally, the investigation extended to other factors such as age, weight and Procrustes distance. However, these parameters also failed to exhibit significant relationships with PC1.

## 4 Discussion

The findings from this study are an essential start to explain the relationships between anatomy, function and breed‐specific adaptations regarding the morphological variation of hyoid bones among dog breeds. There is a significant gap in the reference data on this issue. Researchers can develop hypotheses based on the data from this study for future studies and increase the reference data on this issue.

The observed shape changes in the hyoid apparatus, especially about PC1, suggest that size and shape may not follow a linear relationship. However, the hypothesis that brachiocephalic breeds have a different hyoid bone structure regarding shape variation can be supported.

Brachycephalic breeds such as the French Bulldog and Pug exhibited negative PC1 values, corresponding to a higher and shorter hyoid structure. Previous studies have shown that brachycephalic skull morphology is associated with tongue crowding, altered hyoid‐laryngeal positioning and changes in upper airway dimensions, which may influence tongue mobility and related functions (Plotsky et al. 2016; Seidenburg and Dupre 2021). Conversely, longer nosed breeds, such as the Golden Retriever with positive PC1 values, exhibited different hyoid morphologies that may be more suitable for their specific functional needs.

The lack of significant correlations between shape and factors such as age, weight and Procrustes distance suggests that, although these factors may influence skeletal morphology, they may not be as critical as genetic pressures in shaping the hyoid bone. This highlights the importance of considering each breed's breeding and feeding history when evaluating anatomical structures. Furthermore, the lack of significant findings regarding allometric effects suggests the potential need for further studies with larger sample sizes and a more comprehensive range of breeds to better elucidate the relationships between size, shape and function in the hyoid apparatus.

3D imaging techniques serve as a powerful, non‐invasive approach for enhancing veterinary anatomical research (Bakici et al. 2020; Ekim et al. 2024; Remzi et al. 2019), particularly in the exploration of hyoid bone morphology and its functional implications (Bakici et al. 2019b). For example, these imaging methods have effectively analysed hyoid bone shapes in canine studies. These are crucial for understanding the relationship between anatomy and physiological functions, without interfering with the animals’ natural behaviours or development. In studies involving various dog breeds, advanced imaging can capture intricate details of hyoid anatomy, enabling researchers to make meaningful comparisons that traditional methods might not allow. As imaging technology continues to evolve, the accuracy and feasibility of these techniques in veterinary anatomy are expected to yield even richer insights, from variations in hyoid structure to how these adaptations influence feeding and vocalisation. This adaptability positions 3D imaging as essential in academic exploration and practical veterinary applications. It promotes a deeper understanding of anatomical diversity and its relevance to canine health and behaviour (Agac et al. 2024; Ruzhanova‐Gospodinova et al. 2024; Ünal et al. 2025).

The structural integrity of the hyoid bone is crucial for the coordination of laryngeal closure during the various stages of swallowing, and our study has highlighted the significant morphological diversity of the hyoid bone among different dog breeds (Manchi et al. 2016; Rossi et al. 2013). Notably, we found pronounced shape variations in the hyoid bones of various breeds, particularly in brachycephalic breeds like the French Bulldog and Pug. These breeds exhibited a more compact hyoid structure, as indicated by negative PC1 values, which may suggest a reduced efficiency in their swallowing mechanics. These structural differences could be linked to functional disorders, such as swallowing difficulties, similar to those described in the clinical case referenced. According to Kang et al. (2008), if the hyoid apparatus is malformed or positioned abnormally, it can compromise the synchrony required for effective swallowing, potentially resulting in clinical signs such as dysphagia or regurgitation. It is essential to differentiate whether the morphological variations observed are typical adaptations to breed‐specific traits or indicative of malformations. Given our findings, it is crucial to recognise that the hyoid bones in dogs may exhibit distinct shape variations across breeds, which should be considered when evaluating their anatomical structures. This understanding can facilitate better assessments of swallowing‐related issues in veterinary practice, as the anatomical characteristics of the hyoid bone may significantly influence clinical outcomes. Furthermore, our research emphasises the need for a comprehensive understanding of breed‐specific hyoid morphology to inform diagnostic and treatment strategies for conditions related to swallowing mechanics in dogs.

A study on hyoid malformation in a French Bulldog documented a condition that complicates the management of brachycephalic obstructive airway syndrome, a notable issue in these breeds (De Bruyn and Hosgood 2022). The comparative assessment of hyoid conformation revealed that French Bulldogs exhibited a more acute curvature and increased relative ventrodorsal thickness compared to mesocephalic dogs. Our findings align with these observations, indicating that the hyoid bones in French Bulldogs display a narrower and more ventrodorsally developed structure characterised by negative PC1 values. This similarity underscores the significance of acknowledging the unique hyoid structures among dog breeds, particularly when evaluating pathological conditions. The distinct morphology observed in brachycephalic breeds like the French Bulldog may contribute to the complexities associated with conditions such as brachycephalic obstructive airway syndrome and dysphagia. Recognising these variations is crucial for veterinarians in diagnosing and managing potential health issues related to the hyoid apparatus.

In conclusion, understanding the morphological variation of the hyoid bones in dogs not only contributes to the field of veterinary anatomy but also has practical implications for veterinary medicine, particularly in surgical and rehabilitation practices. Since the hyoid apparatus plays a vital role in basic functions such as swallowing and vocalisation, the information gained from this study may inform clinical approaches to treating conditions associated with hyoid bone morphology. Future research should aim to examine the functional implications of these variations in more depth, potentially leading to improved care and management strategies tailored to the anatomical characteristics of different dog breeds.

## Author Contributions

**Yusuf Altundağ**: conceptualisation, resources, writing – original draft, writing – review and editing. **Ermiş Ozkan**: resources, writing – original draft, writing – review and editing. **Funda Yiğit**: resources, writing – original draft, writing – review and editing. **Kozet Avanus**: resources, supervision, writing – review and editing. **Nedzad Hadziomerovic**: supervision, writing – review and editing. **Tomasz Szara**: supervision, writing – review and editing. **Constantin Spataru**: investigation, writing – original draft.

## Funding

This study was funded by the Tekirdag Namık Kemal University‐ Türkiye Bilimsel ve Teknolojik Araştırma Kurumu.

## Ethics Statement

**Studies involving animal subjects**

This study was approved by the Istanbul University‐Cerrahpaşa, Animal Experiments Local Ethics Committee.

**Studies involving human subjects**

No human studies are presented in the manuscript.

**Inclusion of identifiable human data**

No potentially identifiable images or data are presented in this study.

## Conflicts of Interest

The authors declare no conflicts of interest.

## Data Availability

The original contributions presented in the study are included in the article/supporting information, further inquiries can be directed to the corresponding author/s.
